# Liposome production by microfluidics: potential and limiting factors

**DOI:** 10.1038/srep25876

**Published:** 2016-05-19

**Authors:** Dario Carugo, Elisabetta Bottaro, Joshua Owen, Eleanor Stride, Claudio Nastruzzi

**Affiliations:** 1 Institute of Biomedical Engineering, Department of Engineering Science, Old Road Campus Research Building, University of Oxford, Oxford, United Kingdom; 2 Mechatronics and Bioengineering Science research groups, Faculty of Engineering and the Environment, University of Southampton, Southampton, United Kingdom; 3 Department of Life Science and Biotechnology, University of Ferrara, Italy

## Abstract

This paper provides an analysis of microfluidic techniques for the production of nanoscale lipid-based vesicular systems. In particular we focus on the key issues associated with the microfluidic production of liposomes. These include, but are not limited to, the role of lipid formulation, lipid concentration, residual amount of solvent, production method (including microchannel architecture), and drug loading in determining liposome characteristics. Furthermore, we propose microfluidic architectures for the mass production of liposomes with a view to potential industrial translation of this technology.

The term vesicle is used to denote a supramolecular aggregate composed of amphipathic molecules, commonly natural or synthetic phospholipids, formed in an aqueous phase^1^. “Liposomes” are a particular type of vesicle that are widely employed for biomedical and biotechnological purposes^2^. They are composed of one or more closed shells, or lamellae, consisting of a phospholipid bilayer and enclosing a small volume of aqueous liquid. Their dimensions can vary from tens of nanometres up to hundreds of micrometres depending on the preparation protocol and the final use. Typically, liposomes used in medical applications are unilamellar with an average size of ~100 nm.

The organisation of phospholipid molecules in water is mainly driven by the hydrophobic effect which spatially organises the amphiphilic molecules (phospholipids) so as to minimise entropically unfavourable interactions between hydrophobic acyl chains and the surrounding aqueous phase. Liposomes are extensively used in the cosmetic and pharmaceutical industries as carriers for both lipophilic and water-soluble actives; hydrophilic molecules are entrapped in the interior aqueous compartment, while lipophilic molecules are solubilised into or intercalated within the lipid bilayer. Many hundreds of biologically active compounds, including anticancer and antimicrobial agents, chelating agents, peptides, hormones, enzymes, proteins, vaccines, and genetic materials, have been evaluated for delivery using liposomes^2^.

The first liposomal pharmaceutical product, Doxil^®^ received approval by the Food and Drug Administration (FDA) of the United States of America (USA) in 1995 for the treatment of refractory acquired immune deficiency syndrome (AIDS)-related Kaposi’s sarcoma^3^. To the best of our knowledge, there are about fifteen liposome-based medicines currently approved for clinical usage (see Table S1) and many more are undergoing clinical trials. The administration route of these liposomal drug formulations can be intravenous^4^, intramuscular, epidural^5^, intratracheal or intrabronchial^6^. Other delivery routes have also been investigated (e.g., oral^7^, dermal or transdermal^8^), although they are less frequently employed in association with commercial formulations.

When designing new preparation routes it is important to consider the desirable characteristics of liposomes for specific applications. These are summarised in Table 1, and include the average content of lipids, liposome size, manufacturing method, physical form and excipients, as obtained from the patient information leaflets of 14 commercial medicines containing liposomes (see Table S1 for more detailed information about these formulations and related patient leaflets). For instance, Doxil^®^ is prepared by a multi-step procedure as follows: (a) preparation of multilamellar vesicles (MLVs) by lipid film hydration; (b) formation of large unilamellar vesicles (LUVs) from MLVs by freezing and thawing, followed by liposome extrusion through polycarbonate filters of a defined pore size that results in a homogeneous population of single bilayer vesicles of decreased particle size; (c) establishment of an ion gradient between the external aqueous phase and the interior aqueous compartment of the liposomal preparation (usually by size-exclusion column chromatography); and (d) encapsulation of doxorubicin by active loading^3^.

Over the past few decades different techniques have been proposed for the preparation of liposomes and liposome size, dispersity (Đ)^9^, lamellarity and entrapped liquid volume may all vary depending on the method used.

The majority of the existing methods can be categorised into one of two groups of bulk methods, where the term “bulk” is used throughout this manuscript to describe macroscale or batch techniques.

The first group consists of methods based on the swelling of initially dried preorganised lipid films (i.e. rehydration methods), followed by the mechanical manipulation of the dispersed bilayers^1^. The second group includes methods involving the use of: (i) a cosolvent in which the lipids are soluble, (ii) an additional non-bilayer-forming “coamphiphile”, or (iii) specific ionic species that influence the supramolecular aggregation of the lipids^1^.

More recently, improvements have been made with the development of microfluidic production methods^10^, in which liposome formation occurs within a confined microenvironment. Microfluidic methods have demonstrated potential for achieving higher control over the physical properties of the end product, particularly in terms of liposome size, size distribution and lamellarity. There are however concerns about the viability of this technology on an industrial scale. These concerns arise mostly from the lack of experimental evidence demonstrating: (i) a systematic superiority compared to macroscale bulk methods, (ii) suitability for producing clinically relevant liposome formulations, (iii) efficiency in loading bioactive compounds, and (iv) potential for mass production. In this respect, it should be noted that efforts have been made in the industrial environment to develop controlled precipitation processes for producing liposomes of clinically relevant standards. In this manuscript, we attempt to address these issues by critically analysing state-of-the art microfluidic methods reported in the literature and by performing additional experiments.

## Analysis of Current Techniques

### Liposome preparation: bulk versus microfluidic methods

Microfluidic approaches to liposome production include electroformation and hydration^11^, extrusion^12^, pulsed jetting^13^, double emulsion templating^14^, ice droplet hydration^15^, transient membrane ejection^16^, droplet emulsion transfer^17^ and hydrodynamic focusing^18^. Recently, a microfluidic method based on the hydrodynamic pinch-off mechanism has also been proposed for producing monodisperse, cell-sized (5–20 *μ*m) unilamellar liposomes with excellent encapsulation efficiency^19^.

Many of the aforementioned techniques however result in the production of relatively large vesicular systems (i.e., Giant or Large Unilamellar Vesicles) or microtubules, limiting their potential for the production of nanoscale drug delivery vehicles. Furthermore, being simply a miniaturised version of their analogous bulk counterparts, many of the aforementioned methods cannot be regarded as strictly microfluidic. However, among these, the microfluidic hydrodynamic focusing (MHF) approach presents the typical physical characteristics of microfluidic systems (i.e., including low Reynolds number and diffusion dominated mass transfer) and, to the best of the authors’ knowledge, represents the only viable microfluidic method for producing lipid-based nanoscale vesicular systems with potential for clinical application.

Only limited attention has been devoted to this technique in previous publications^5^ however, and a critical evaluation of the method has not been carried out.

The MHF method relies on the use of microfluidic devices with a cross flow geometry or, more rarely, with a 3D annular coaxial geometry^20^ (see Table 2 for a comparative analysis of the MHF methods)^21^,^22^,^23^,^24^,^25^,^26^,^27^,^28^,^29^,^30^.

Typically, a stream of lipid in alcohol solution is forced to flow in the central (or inner) channel of the device. The lipid stream is intersected and sheathed by two lateral (or coaxial) stream(s) of a water phase (typically distilled water or aqueous buffers). In this way, the lipid containing stream is hydrodynamically focused into a narrow sheet having a rectangular cross-section in the case of microchips with cross flow geometry, or a circular cross-section in the case of 3D annular coaxial chips. Notably, the size of the focused stream can be tuned by adjusting the volumetric flow rate ratio (FRR) between the lipid and water phase streams, and the total flow rate (TFR)^21^.

The formation of liposomes in MHF chips is governed by the diffusion of different molecular species (mainly alcohol and water, but also lipids) at the liquid interface between the solvent (alcohol) and non-solvent (water)^22^,^31^ phases. The alcohol in which the lipids are initially solubilised diffuses into the water (and concomitantly the water diffuses into the alcohol) until the alcohol concentration decreases to a critical level, below the solubility limit of the lipids. As such, the alcohol diffusion triggers the formation of liposomes by a mechanism described as “self assembly”^21^,^22^. Specifically, it is believed that the reciprocal diffusion of alcohol and water across the focused alcohol/water interface causes the lipid to precipitate, resulting in the formation of intermediate structures, in the form of oblate micelles, that subsequently form liposomes^22^.

MHF microfluidic techniques have been shown to produce uniformly dispersed liposomes and allow for direct control of liposome size *via* fine adjustments to either FRR or TFR. Jahn *et al.* postulated that: “decreasing the sample stream width to micrometre length scales allows for controlled and reproducible mechanical and chemical conditions across the stream width, especially compared to more traditional bulk-phase preparation techniques (i.e., test tubes and beakers)”^21^. In discussing bulk-phase production methods the authors referred to a number of procedures which could not be clearly identified^21^. In addition, van Swaay and DeMello further reinforced this concept in a recent, comprehensive review article by stating that “the microfluidic flow-focusing method of vesicle production has no analogous protocol on the macroscale” (i.e. bulk method)^10^.

It is often accepted that bulk methods are associated with heterogeneous and poorly-controlled chemical and mechanical conditions during lipid vesicle formation, resulting in non-uniform vesicles in terms of both lamellarity and size. For these reasons, additional post processing steps are often required for obtaining homogeneous vesicle suspensions. These processes include: (a) vesicle extrusion through the pores of polycarbonate membranes, (b) treatment with ultrasound, or (c) repetitive freezing and thawing^1^. In contrast, being characterised by laminar flow conditions and diffusive mass transfer, MHF has the ability-at least in theory-to produce liposomes with excellent control over size and lamellarity. Furthermore, microfluidic technology offers additional advantages over bulk methods, including the possibility of *in situ* monitoring of the liposome formation process, continuous production and scaling up by microreactor parallelisation^31^.

### Effect of microfluidic parameters on liposome characteristics

A number of investigations of the effect of microfluidic parameters on liposome characteristics have been carried out in recent years^7^,^8^,^9^,^10^,^11^,^12^,^13^,^14^,^15^,^16^ and the main findings can be summarised as follows: (a) the mean diameter of the liposomes produced is directly related to lipid concentration^25^,^26^,^27^ and inversely related to FRR^21^,^22^, (b) the TFR appears, in contrast, to have only a small effect on liposome size^21^,^22^. However, limited comparative data are available on the effect of microdevice geometry (i.e. different channel patterns) on liposome characteristics. For instance, Jahn *et al.* used two similar microfluidic devices to study the combined effect of device geometry and hydrodynamic flow focusing regimes on liposomes. They used MHF chips differing in the width of the mixing channel (10 μm and 65 μm, respectively), and concluded that similar liposome size distributions were obtained under different hydrodynamic flow focusing conditions; for instance, at high FRR = 24 and 36, the liposomes produced by the two MHF devices were quite similar: 31 vs 38 nm and 27 vs 32 nm in diameter for the 10 and 65 μm devices, respectively^22^. Hood and DeVoe investigated the effect of channel aspect ratio (i.e., height:width ranging from 0.5:1 to 100:1) in traditional flow focusing geometries, and concluded that high aspect ratio devices resulted in more uniform liposomes and higher production rates^32^.

Alternative microfluidic architectures have been recently proposed by the Perries’s group, which consisted of Y-shaped mixers incorporating staggered herringbone elements to induce chaotic advection^29^. Unfortunately, no direct comparison between these alternative architectures and conventional MHF chips was reported. Concentric capillary arrays have been recently developed by Hood and co-authors and employed as a cost-effective and facile route to liposome production using three-dimensional flow focusing. Liposomes produced with this method had comparable size and lower size dispersity compared to those obtained using traditional MHF chips^20^.

### Open questions on liposomes by microfluidics

In spite of the promising opportunities offered by microfluidics for liposome preparation, a number of questions still remain to be answered (see points i-vi below). Notably, such issues are also relevant to the microfluidic-based production of other nanoparticulate systems, such as polymeric micelles and niosomes^31^,^33^.

(i) In the literature, no comparison between liposomes produced by bulk methods and microfluidics is provided. This has been previously attributed to the fact that MHF methods don’t have analogous protocols at the macroscale^5^. Notably, methods based on the use of mixers, which are routinely employed in the industry, may not represent a scaled up version of microfluidic flow focusing regimes.

Moreover, liposomes obtained by microfluidic MHF at various FRRs differ in terms of both lipid and alcohol content; therefore their dimensions cannot be directly compared. It is indeed well known that lipid content greatly influences the dimensions of liposomes, as evident from ethanol injection bulk methods^34^.

(ii) The coating material studied is often limited to the anionic mixture DMPC/cholesterol/dihexadecylphosphate (DHP)^22^,^21^; limited data is provided on the comparison between different lipid mixtures or on the use of cationic lipids^29^, which may be more suitable for medical or biotechnological applications (e.g. as delivery systems for anticancer drugs or as transfection reagents), and which are routinely used in the industry.

(iii) The lipid concentration typically used for liposomes produced by microfluidics is relatively low with respect to liposomes present in commercial medicines (see Table S1); for instance, in the case of liposomes produced with a FRR = 10 or 30, the typical final total concentration of lipids ranges between 0.16 and 0.45 mM^11^,^12^,^13^,^14^,^15^,^16^,^17^,^18^,^19^,^20^,^21^.

In other studies, the concentration of lipids in the final liposome suspension typically ranges between 0.1 to 2.0 mM, depending on the FRR. This limitation of MHF techniques is particularly critical when liposomes produced by microfluidics are compared with those present in commercially available medicines, where the lipid concentration in the ready-to-be-administered suspensions typically ranges between 5 and 25 mM^3^,^7^.

(iv) In the majority of reports isopropanol (IPA) is used as the lipid solvent^21^,^23^,^24^,^25^; however, no data is provided on the residual IPA content. Only few studies explore the use of ethanol as a less toxic alternative for medicinal applications, which would also comply with routine industrial processes^35^.

Most importantly, to the best of the authors’ knowledge the effect of residual alcohol content on light scattering measurements (generally used to determine the size of vesicles) has not been taken into consideration in the large majority of previous studies. Given that the viscosity of water/alcohol (isopropyl alcohol or ethanol) varies in relation to the alcohol content in the final liposome suspension (and thus on the FRR employed), a systematic determination of fluid viscosity at varying FRR regimes may be required to obtain accurate dimensional measurements. Notably, small variations in fluid viscosity may be sufficient to significantly affect light scattering measurements^36^. Although this effect could be reduced by significantly diluting the liposome suspension prior to dimensional analysis, this may not be applicable to many MHF techniques which suffer from a relatively low liposome concentration in the end-product, particularly when operated at high FRR.

(v) To the best of our knowledge, only a few reports are available that consider the effect of different chip architectures (i.e. channel dimension and angle between the main and side channels) on liposome characteristics^22^. Other studies have focused on the effect of channel aspect ratio^32^ in conventional flow focusing geometries.

(vi) Finally, for liposomes produced by microfluidics, there has been little or no systematic characterisation of drug encapsulation efficiency or the effect of drug encapsulation on liposome size.

## Results and Discussion

### Design principles for MHF chips

In the present study, different device architectures have been considered for liposome production. Devices include microscale chips (cross-sectional dimensional range of 100–320 μm) with mixing channel displaying distinct architectural features (i.e., straight, serpentine-like, and containing micropillar structures) and scaled-up versions of microscale flow focusing architectures, with cross-sectional channel dimension in the millimetre range. Considering the spectrum of different architectures described in the literature, the following general principles have been considered in this study for designing and constructing chips employed for liposome production.

(i) *Chip dimensions*. The total width and length of the chip should be defined to comply with the dimensional constraints imposed by the typical size of microscope stages that are routinely used to assess the correct functioning of the chips. As a general design principle, the microchannel architecture should fit within the maximum width and length of conventional glass slides for microscopy (i.e., width = 25–50 mm and length = 75 mm). At the same time, the thickness of the device should comply with the working distance of microscope objectives. It should be noted that whilst *in situ* microscope observation may not be required in an industrial setting, we consider it as a desirable requirement at a research and development stage.

(ii) *Channel dimensions*. The inlet channels should be long enough to allow the fluid flows to fully develop before they intersect with each other, allowing for a stable and predictable flow focusing regime. Channels should also be designed so as to guarantee sufficient spacing between the inlet ports, allowing for robust and practical connection with tubing and pumping units. A typical inlet channel length for MHF chips is in the range of 5–10 mm; note that longer channels result in higher backpressure, which could potentially compromise device usability. Finally, the mixing channel should be designed to allow for complete mixing of the solvents (ethanol or isopropanol, and water) at the selected operating flow rates, and to comply with the dimensional requirements imposed by microscope interfacing.

(iii) *Relative orientation of the inlet channels.* In MHF chips, the angle between the side and central inlet channels should be defined so as to minimise fluid dynamic perturbations at the intersection between flows, particularly if devices are designed to operate at high throughput regimes. An angle in the range 30°–60° was deemed suitable for the applications described in the present study.

(iv) *Materials*. The constitutive materials should satisfy different requirements, including resistance to solvents, optical transparency, compatibility with microfabrication and bonding techniques, low surface roughness, and commercial availability. In this specific study, PDMS has been identified as the optimal material choice, given its compatibility with pharmaceutical-grade solvents (i.e., ethanol), ease of surface treatment by exposure to oxygen plasma, ability to conform to a surface, and potential for bonding with commercially available glass slides.

(v) *Fabrication method*. Traditional soft lithographic methods based on SU-8 moulding can be employed for producing PDMS microchannel architectures, which can be irreversibly coupled to glass substrates using plasma bonding techniques. Notably, glass is chemically inert and its surfaces smoothness is suitable for optical microscopy. With an aim to develop cost-effective and facile microfabrication methods with potential for large-scale chip fabrication, we also employed an in-house developed technique which combines micromilling with epoxy-based replica moulding. Both these methods allow for obtaining well-defined cross-sectional channel features, which may be complex to achieve using wet etching techniques.

(vi) *Operating regime*. Microfluidic flow focusing was selected as a microfluidic-based regime for nanoscale liposome production, and its performance compared with other micromixing geometries. Notably, MHF operated at laminar flow conditions has been previously demonstrated to allow for precise control over the interfacial boundaries between solvent and co-solvent streams, resulting in diffusion dominated mass transfer and leading to liposomes of relatively uniform physical properties. Furthermore, liposome size and dispersity in MHF chips has the advantage of being potentially controlled on-demand by finely adjusting the hydraulic boundary conditions. MHF devices were compared with other micromixing geometries, including those containing serpentine-like microchannels and micropillar arrays.

### Production of liposomes using different microfluidic architectures

**Figure 1:**
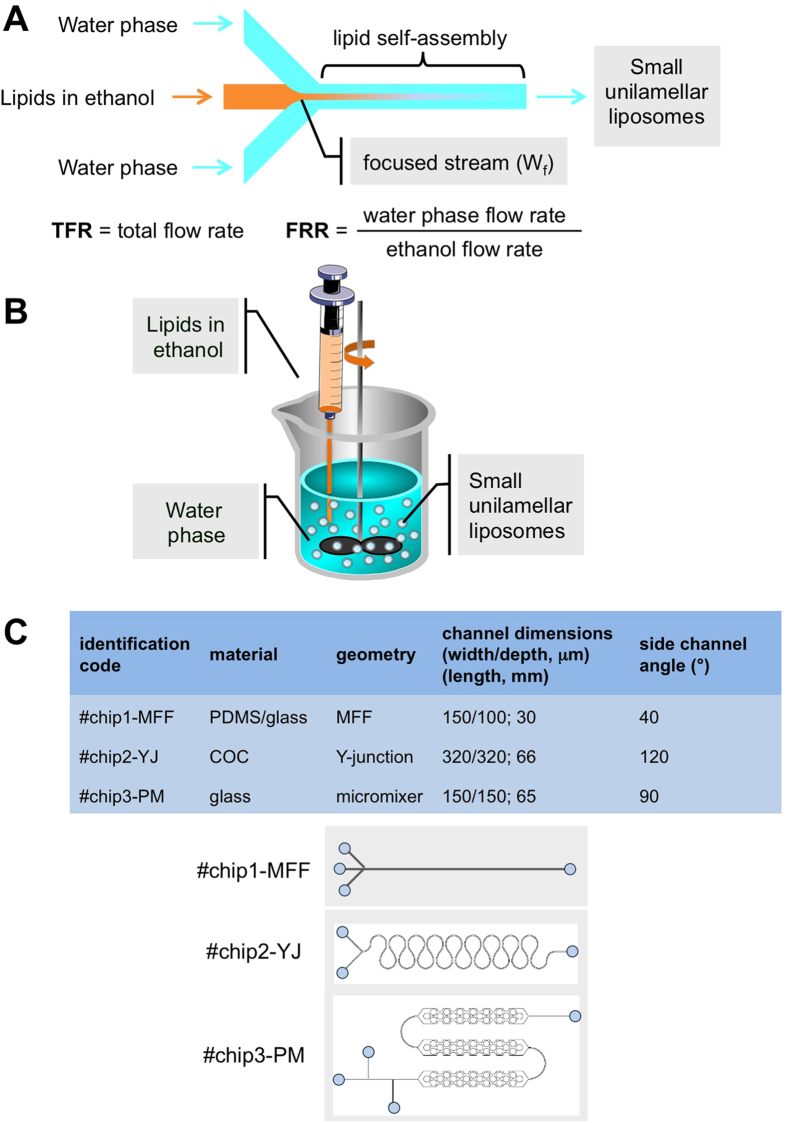
Schematic representation of the process of liposome formation. Panel A shows a schematic representation of a microfluidic device, namely a MHF microchip (#chip1-MHF), and the process of liposome (SUV) self-assembly. Panel B shows a schematic representation of the ethanol injection procedure. Panel C illustrates the geometrical characteristics of the chips employed for the microfluidic experiments. #chip1-MHF comprises three inlet microchannels with 30° intersection angle between each other, and a mixing microchannel having width, depth and length of 150 μm, 100 μm and 30 mm, respectively. #chip2-YJ comprises two inlet microchannels with 120° intersection angle between each other, and a mixing microchannel having width, depth and length of 320 μm, 320 μm and 66 mm, respectively. #chip3-MP comprises three inlet microchannels with 90° intersection angle between each other, and a mixing microchannel with three pillar mixing elements, having width, depth and length of 150 μm, 150 μm and 65 mm, respectively. Teflon^TM^ tubes were used to connect the microfluidic platform with syringes. The volumetric flow rate was controlled using syringe pumps.

In the remainder of this manuscript, the “real” advantages of microfluidics over bulk methods are discussed in order to address the issues reported above. Specifically, the production of liposomes employing different microfluidic chips (see Fig. 1) and lipid formulations was evaluated in terms of the size and homogeneity of the end product, with particular emphasis on the potential industrialisation of microfluidic methods.

Moreover, a detailed analysis is provided of the effects of the operating parameters (especially FRR) on liposome dimensions, taking into consideration the effect of residual alcohol on the viscosity of the liposomal samples and thus on the determination of liposome size by light scattering measurements. Liposomes produced using MHF were compared with those obtained by the bulk ethanol injection method.

Ethanol injection was selected as a bulk technique since, in our opinion it strongly resembles MHF liposome formation (in terms of simplicity and physical conditions) relative to other bulk techniques. Notably, this method results in the formation of small unilamellar vesicles (SUV); therefore it does not require post-processing homogenisation steps. Similar to many microfluidic protocols, phospholipids are firstly dissolved in ethanol; a small amount of the lipid solution is then injected into water, above the transition temperature (T_m_), triggering SUV formation.

Analogously to MHF, the formation of liposomes is ascribed to the miscibility of ethanol and water that causes the diffusion of alcohol molecules into water and the consequent “self assembly” of lipids to form liposomes. The size and homogeneity of the SUVs is dependent upon the experimental parameters, specifically: lipid concentration, rate of injection of the alcoholic lipid solution and stirring rate^1^. In this study an injection method, more strictly resembling microfluidic approaches, was employed. The lipid mixture was injected by a syringe pump at a constant flow rate of 500 μL/min and the water phase was maintained under mechanical stirring by a three blades impeller, at 300 r.p.m. In addition, bulk experiments were conducted varying both the quantity of lipid (from 0.86 to 4.0 mM) at a fixed ethanol content, and the ethanol content (from 1.9 to 9.0%, v/v) at a fixed quantity of lipid (see Table S2).

**Figure 2:**
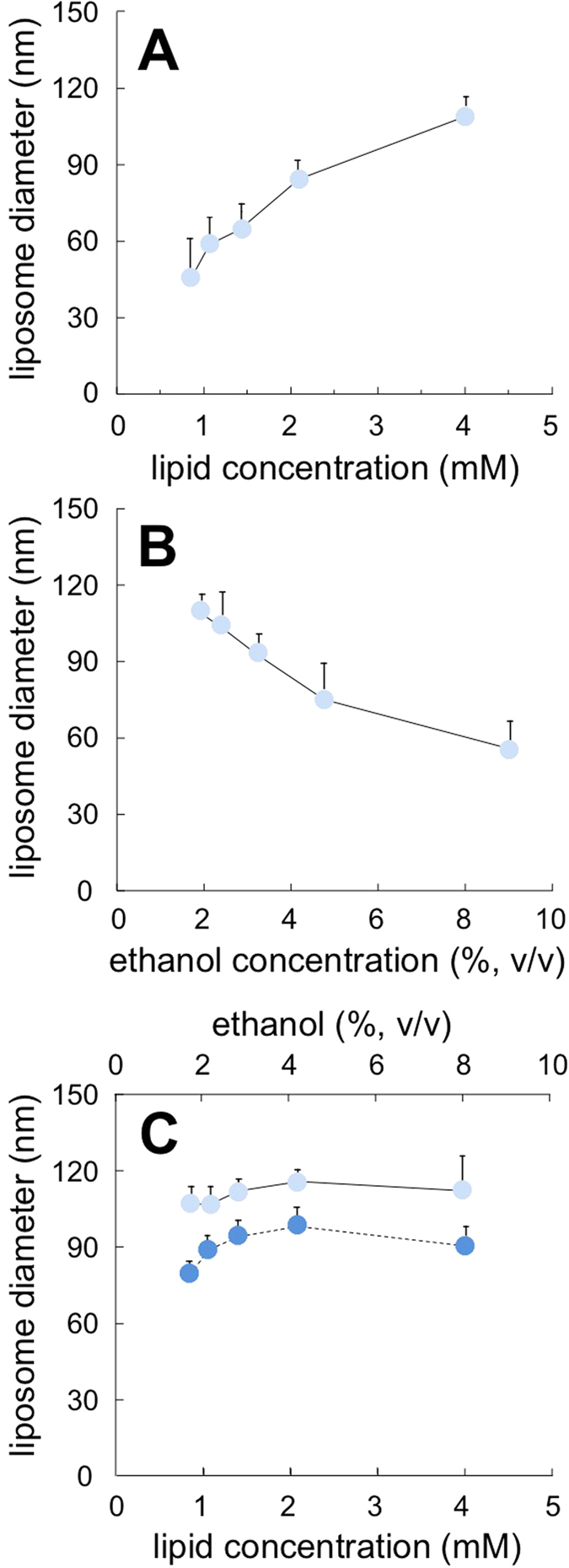
Effect of the variation of lipid concentration (**A**), ethanol content (**B**) and simultaneous variation of both parameters (**C**) on the dimension of liposomes produced by ethanol injection. For comparison, data relative to liposomes prepared by MHF microfluidics are also reported in panel C (dashed line). Data correspond to the Z-average, determined by DLS. Liposomes were constituted of PC/cholesterol 4.0−0.4 mM, and data represent the mean of three independent samples, measured in triplicate ± S.D.

Data reported in Fig. 2, referring to liposomes produced by the “controlled” injection method, show that the concentration of ethanol and lipid significantly affected the dimensional characteristics of liposomes. For instance, upon increasing the lipid concentration a concomitant size increase of the liposome (Fig. 2A) was observed. The liposome mean diameter was equal to 46 ± 9 nm and 109 ± 5 nm at a lipid concentration of 1 mM and 4 mM, respectively (data correspond to the Z-average, determined by DLS, and are the mean of three independent samples, measured in triplicate ± S.D.). The ethanol content had the opposite effect; the progressive increase from 2% to 9% (v/v) caused a decrease in liposome size (Fig. 2B). Finally, in Fig. 2C the effect of the concomitant variation of lipids and ethanol content is reported. In this case, the two effects counterbalanced each other; in fact liposomes maintained an almost constant size of ~115 nm. For comparison, the size of liposomes produced by MHF is also reported (dashed line). Notably, liposomes produced by MHF were smaller than those produced by the bulk method, although the overall dimensional trend was similar.

**Figure 3:**
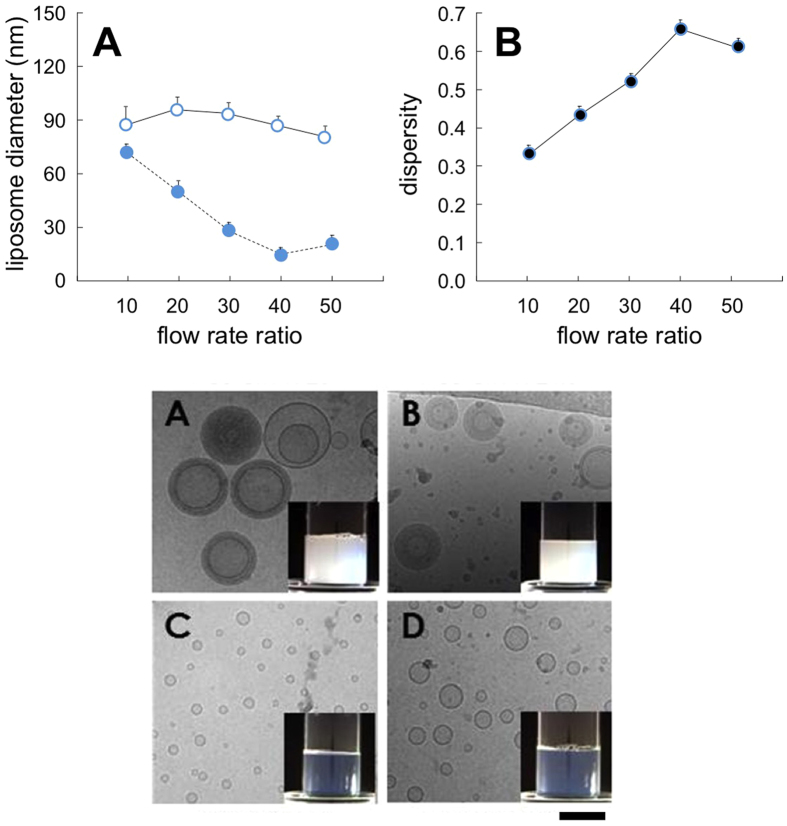
Effect of the variation of lipid composition on the size (upper part, panel A) and dispersity (upper part, panel B) of liposomes produced by MHF microfluidics. Liposomes were constituted of PC/DDAB 9.0−1.0 mM (filled circles, dashed line) or PC/cholesterol 9.0−1.0 mM (open circles, plain line). Data correspond to the Z-average, determined by DLS, and are reported as the mean of three independent samples, measured in triplicate ± S.D. In the lower part, cryo-TEM and macroscopic aspect (insets) of empty PC/cholesterol (**A**) and PC/DDAB (**C**) are reported. For comparison, images of the corresponding ivermectin loaded liposomes are also reported (**B,D**). Bar corresponds to 100 nm.

With respect to the lipid formulation, the results reported in Fig. 3 strongly suggest that, as for bulk procedures, the lipid composition plays a fundamental role in determining the size of liposomes produced by microfluidics. It is evident that the use of a cationic lipid mixture resulted in a marked decrease in liposome size when compared to a neutral lipid formulation. For instance, at FRR = 30 neutral liposomes had an average size of 87 ± 4 nm compared to 28 ± 2 nm for cationic liposomes (data correspond to the Z-average, determined by DLS, and are the mean of three independent samples, measured in triplicate ± S.D.). The macroscopic and microscopic appearance of the produced liposomes is also reported in Fig. 3.

Importantly, our microfluidic experiments demonstrate that small and uniform liposomes can be produced using relatively high concentrations of lipids. The final concentration of lipids was indeed comprised between 9.0 mM (at FRR = 10) and 3.2 mM (at FRR = 30), which corresponds to 6.8 mg/ml (at FRR = 10) and 2.4 mg/ml (at FRR = 30). Notably, the total concentration of lipids (expressed in mg/ml) in liposomes present in medicines ranges between 0.1 mg/ml (for Epaxal^®^) and 103 mg/ml (for Marqibo^®^), and typically falls within the 5–15 mg/ml range for the majority of formulations (see Table S1). It can therefore be concluded that microfluidic methods are potentially suitable for the preparation of liposomal suspensions to be included in commercial, pharmaceutical formulations. Moreover, in order to minimise the content of toxic constituents we demonstrate that ethanol is a suitable substitute for the more toxic isopropanol in microfluidic experiments, particularly in view of a potential translation of this technology into the industrial environment.

**Figure 4:**
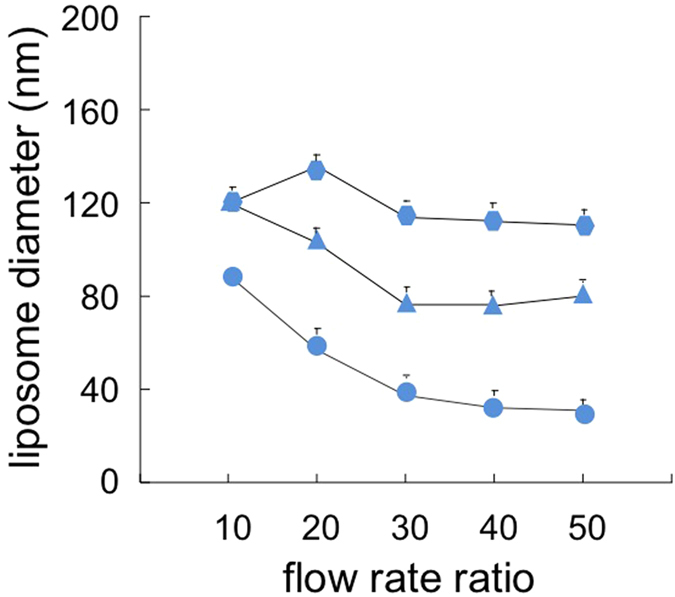
Comparative analysis of liposomes produced by microfluidic technique with different microchips. Data refer to Z-average of liposomes produced by #chip1-MHF (circles), #chip2-YJ (hexagons) or #chip3-PM (triangles). Liposomes were constituted of PC/DDAB 9.0−1.0 mM, produced at the indicated FRR and TFR = 37.50 μl/min. Data are reported as the mean of three independent samples, measured in triplicate ± S.D.

To evaluate the potential effect of chip geometry and material properties on liposome preparation, three different microfluidic devices were constructed; each having significantly distinct design parameters (see Fig. 1). Our results, shown in Fig. 4, indicate that liposome size and size distribution are not only functions of flow rate ratio, but are also influenced by the device size and architecture. For all chips, the increase in FRR caused, as expected, a progressive decrease of the liposome dimensions; liposomes prepared with #chip1-MHF were smaller than those prepared with all other chips. In particular, the liposomes obtained by #chip1-MHF were in the range 30 and 80 nm, depending on FRR. This effect was tentatively attributed to the fact that #chip1-MHF has the smallest channels and the MHF geometry has two interfaces for ethanol diffusion, whilst #chip2-YJ has only a single surface of boundary between the water phase and the lipid ethanol solution. Potential establishment of secondary flows (i.e. Dean flows) within curved channels (present in both #chip2-YJ and #chip3-PM) may have influenced on mass transport of solvent and co-solvent in these geometries, and therefore also the obtained liposome size. Furthermore, differences in the total area available for mixing across different chips is also likely to have influenced the physical properties of the end-product.

**Figure 5:**
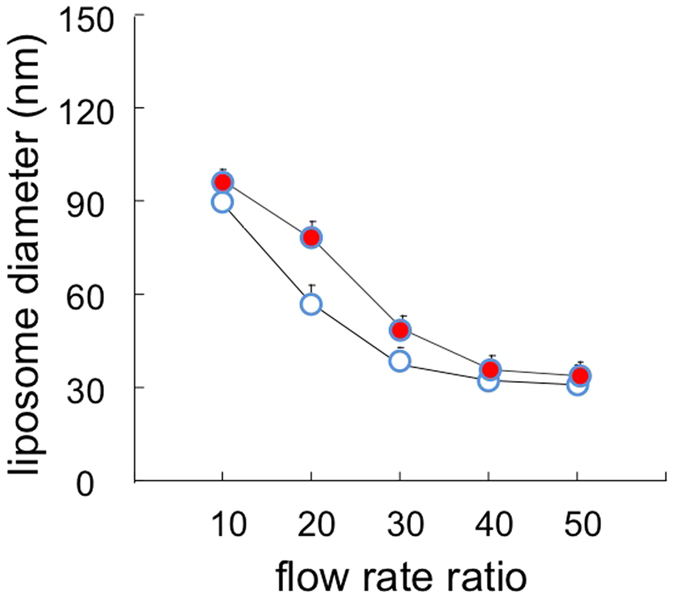
Effect of the encapsulation of 0.1 mM ivermectin on the size of microfluidic produced liposomes (filled circles). For comparison, the size of empty (water-filled) liposomes is also reported (open circles). Liposomes were produced by #chip1-MHF at TFR = 37.50 μl/min and FRR = 30. Data are reported as the mean of three independent samples, measured in triplicate ± S.D.

Finally, the effective encapsulation of drugs by liposomes produced using MHF was investigated. Ivermectin was employed as a model drug since it has recently been shown to be a highly potent inhibitor of yellow fever virus replication and, although less efficiently, of several other flaviviruses. Figure 5 shows that this drug did not cause large modification of the liposome size produced by MHF microfluidics, as the liposome containing ivermectin were only marginally larger than the empty ones. Notably, the encapsulation efficiency of ivermectin in liposomes was extremely high, exceeding 95%. All together, the favourable characteristics of ivermectin loaded liposomes could be potentially ascribed to the physico-chemical properties of the drug molecule (i.e. size, charge, solubility etc.).

### Industrially applicable microdevices

**Figure 6:**
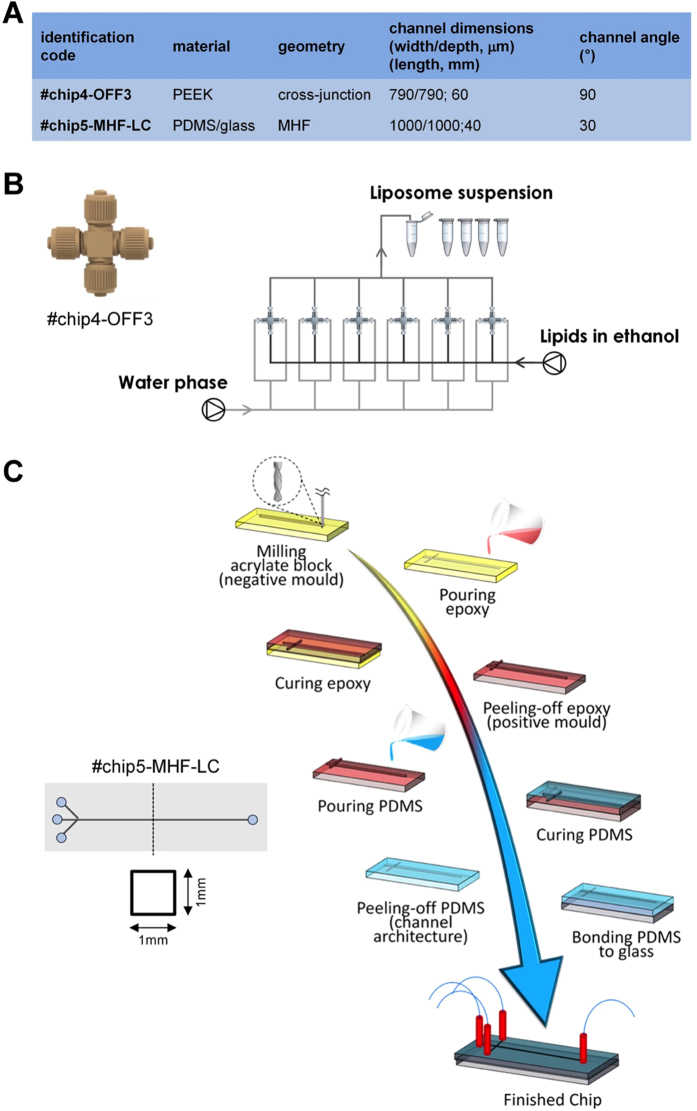
(**A**) Geometrical characteristics of easy-to-build chips for liposome production. (**B**) Schematic of *#chip4-OFF3*, and potential parallelized network for mass production of liposomes. (**C**) Schematic of *#chip5-MHF-LC* and related fabrication method developed in house (μMi-REM).

Much remains to be proven about the industrial applicability of microfluidics, especially in the search for methods (microdevices) which are able to fulfill the different requirements of clinically relevant formulations. In this respect, the development of microfluidic-based technology with clear advantages over bulk methods represents an ongoing challenge. A possible way to bring microfluidic techniques to an industrial level would be the development of easy-to-handle microfluidic chips based on: (a) cost-effective microfabrication technologies or (b) “off-the-shelf” components that are commonly employed for high performance liquid chromatography (HPLC) procedures. In a series of experiments we demonstrated that both #chip4-OFF3 and #chip5-MHF-LC (depicted in Fig. 6) represent interesting examples of easy-to-build MHF chips for liposome production. Particularly, #chip5-MHF-LC represents a scaled-up version of conventional MHF architectures, with channel cross-section of 1 mm × 1 mm and a 30° intersection angle between central and side inlet channels. Chips were produced using μMi-REM, a facile and cost-effective replica moulding technique developed in house (see Fig. 6C)^21^. The method relies on the following steps: (i) milling of the microchannels in a block of acrylic (negative mould); (ii) pouring of low-cost epoxy adhesive resin over the milled acrylic block and curing; (iii) decoupling of the solidified resin layer from the acrylic block (positive mould), pouring of liquid PDMS over the epoxy layer and curing; finally (iv) plasma bonding of the PDMS layer to a glass surface. With this method, microscale architectures can be fabricated without resorting to advanced technological equipment or laborious and time-consuming intermediate procedures.

**Figure 7:**
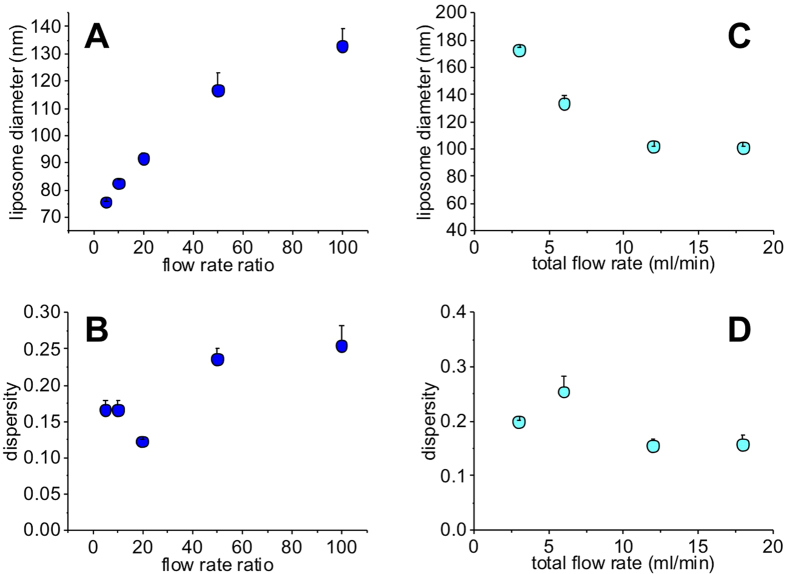
Size and size distribution of liposomes produced by high-throughput microfluidic architecture with scaled up channel dimension (#chip5-MHF-LC). (**A,B**) Dependence of liposome mean size (**A**) and dispersity index (**B**) on the flow rate ratio (FRR, ranging from 5 to 100) at a fixed TFR of 6 ml/min. (**C,D**) Dependence of liposome mean size (**A**) and dispersity index (**B**) on the total flow rate (TFR, ranging from 3 to 18 ml/min) at a fixed FRR of 100.

In order to directly compare our results obtained using #chip5-MHF-LC with those obtained using other MHF architectures reported in the literature, we selected a lipid formulation employed in a recent comparative study by Hood and Devoe, in which three different MHF devices were tested and characterised^22^. Particular attention was also devoted to the selection of a lipid mixture suitable for pharmaceutical applications and resembling the composition of liposome containing medicines. Figure 7 shows the mean size and dispersity index of liposomes obtained using this device. Note that the term “dispersity” is used in this manuscript as an alternative to “polydispersity”, to comply with IUPAC recommendations^9^.

Despite the large channel dimension, compared to previously reported microfluidic devices, liposomes obtained using #chip5-MHF-LC had a mean size and dispersity comparable to conventional microfluidic approaches (Fig. 7A,B). Importantly, we demonstrated the production of clinically relevant liposomes, in terms of size, lipid composition and content (see specific considerations reported in the previous paragraph), at TFR of up to 18 ml/min. Notably, this flow rate is significantly higher than any other value reported in studies describing microfluidic techniques (see Table 2). Liposome size was observed to reduce with increasing TFR (at a fixed FRR of 100), while increasing FRR resulted in increased liposome size. This finding appears to be contrary to previous results obtained using different microscale geometries and therefore merits further investigations (i.e., particularly on the fluid dynamic and mass transport phenomena occurring within these scale-up architectures).

Finally, we demonstrated that #chip4-OFF3 assembled from off-the-shelf components produced liposomes with small dimensions (90–150 nm) and uniform distribution (Đ < 0.3) (data not shown). Notably, the use of “off-the-shelf” components could allow an easy implementation of parallelised networks (see Fig. 6 for a suggested configuration) for mass production of liposomes (or other colloidal assemblies intended for consumables). Being produced at an industrial scale, these components also present low inter-sample variability which is of particular importance for parallelisation purposes.

Despite the potential advantages offered by the proposed microfluidic systems, a more pervasive understanding of the effect of the microfluidic architecture on liposome characteristics may be beneficial to optimise device performance and their scaling-up for mass production of liposomes. This will represent the subject of future investigations.

## Concluding Remarks

The potential for microfluidic-based technology to produce nanoscale lipid vesicles for medical applications has already been demonstrated. However, the translation of this method to the industrial environment has been hindered by several limiting factors. In this paper, we critically review the state-of-the-art microfluidic methods and perform additional experiments in an attempt to address the associated research questions. The following concluding remarks can be drawn from our studies:

(i) A suitable bulk counterpart to MHF microfluidic methods has been identified (referred to as “controlled ethanol injection”), which makes it possible to carry out a systematic comparison between the two methods. Using this technique, we have demonstrated that liposomes produced using microfluidics were smaller and more uniform in size than the ones produced by controlled injection, whilst no appreciable differences in liposome dimensional stability were found over time (data not shown). MHF thus has the potential advantage of being able to generate a wider range of mean liposome sizes, whilst maintaining lower size dispersity compared to bulk counterparts. Furthermore, it allows for flexible, application-specific tuning of liposome size through changes of the hydrodynamic boundary conditions.

(ii) Both lipid and ethanol concentration have a significant effect on liposome properties (in both bulk and microfluidic methods). This is of particular importance when results taken at different FRRs are compared and interpreted. Notably, changing FRR not only causes changes in the fluid dynamic field (i.e., width of the focused stream) but also in the physico-chemical properties of the fluidic environment.

(iii) The lipid formulation plays an important role in determining liposome properties. Specifically, charged lipids generated smaller vesicles compared to uncharged lipids, under identical experimental conditions. Importantly, MHF microfluidic methods were also found to be suitable for producing different liposome formulations.

(iv) Microfluidic methods are suitable for producing small and uniform liposomes at relatively high concentrations of lipids and using solvents with relatively low toxicity (i.e., ethanol). This has important implications for the potential utility of this technology in the pharmaceutical industry. Furthermore, compared to bulk methods, no post-production processing (i.e., sonication, extrusion, freezing and thawing) is required with MHF methods to obtain liposomes of desirable characteristics.

(v) It is possible to efficiently encapsulate biologically active compounds in liposomes produced using microfluidics. Notably, on-chip liposome loading with bioactive compounds (both hydrophilic and lipophilic) has seen a limited number of advancements in recent years, and may represent an exciting avenue of research in the near future.

(vi) High-throughput and easy-to-build microfluidic architectures can be constructed for mass production of liposomes, without significantly influencing the quality of the end-product compared to conventional MHF devices. In this study, we have demonstrated liposome production at volumetric flow rates of up to 18 ml/min.

## Methods

### Chemicals

Highly pure phosphatidylcholine (PC) 90% from soybean was purchased from Phospolipon 90G Lipoid, Germany; cholesterol 97% from Fluka, Germany; and dimethyldioactdecyl-ammoniumbromide (DDAB) from Sigma-Aldrich, UK. 1,2-dimyristoyl-sn-slycero-3-phosphocholine (DMPC) and 1,2-dimyristoyl-*sn*-glycero-3-phosphoethanolamine-N-[methoxy(polyethylene glycol)-2000] (ammonium salt) (14:0 PEG2000 PE) were purchased from Avanti Polar Lipids; polydimethylsiloxane (PDMS) monomer Sylgard^®^ 184 and curing agent were purchased from Dow Corning (USA); SU-8 photoresist was obtained from Chestech (UK); trichloro (1 H,1 H,2 H,2 H)-perfluorooctylsilane, hydrofluoric acid, ammonium fluoride, ivermectin, and suramin sodium salt were purchased from Sigma-Aldrich (UK). All the other regents and solvents not included in this section were also purchased from Sigma-Aldrich (UK).

### Microfluidic device fabrication

#### “Cross-flow” chip

The “Cross-flow” #chip1-MHF consists of polydimethylsiloxane (PDMS) and glass layers, and was produced with conventional soft lithography techniques. Firstly, an SU-8 mould with the designed microchannel pattern, consisting of three inlets and one reaction channel, was prepared following the standard lithography protocols. Subsequently, the mould was covered with a layer of a 10: 1 (w/w) PDMS precursor and curing agent mixture and heated for 1 h at 80 °C for the polymer to cure. The PDMS sheet with the microchannel architecture fabricated on the surface was then removed from the mould and permanently bonded to a glass slide after oxidizing its surface *via* plasma treatment.

#### “Y-junction” chip

The “Snake mixer slide” #chip2-YJ was obtained from Thinxxs, Germany. The mixer was integrated on a microscope slide made of cyclic olefin copolymer (COC).

#### “Three-inlets pillars” chip

For the fabrication of the “three-inlets pillars” #chip3-PM, a photolithography/wet etching procedure was used. Briefly, the channel network was designed using AutoCAD drawing software first. A film negative of the desired final size was then prepared by a commercial photo mask producer to form the optical mask. Crown white glass plates (thickness of 1.5 mm) coated with a thin layer of chromium metal mask plus an upper layer of positive photoresist were used for channel network fabrication. With UV exposure, the pattern of interconnecting channels was transferred from the negative film to the photoresist layer on the glass which was then developed and removed, together with the chromium layer, to reveal the channel areas of the glass to be etched. The glass plate was baked in an oven at 80 °C overnight to dry and harden the mask on glass. The channels were then etched using 1% hydrofluoric acid buffered with 5% ammonium fluoride solution at 65 °C, under ultrasonic agitation (Ultrasonic Cleaner, VWR, UK). Finally, the etched glass was thermally bonded (595 °C for 3 h) with a top plate of the same material into which outlet and inlet holes had been previously drilled. Prior to the bonding step, the cross-sectional profile of the etched microchannel was measured using a surface profilometer.

### Liposome preparation (bulk methods)

#### Ethanol injection

The ethanol injection method was applied to prepare liposomes. The required quantities of lipids were dissolved in ethanol: PC 90G (40–90 mM), cholesterol (4–10 mM), DDAB (10 mM). The resulting organic solution (0.5 ml) was then injected by a syringe pump (KD scientific, New Era pump System) at flow rate of 500 μl/min under magnetic stirring (300 rpm) in an appropriate volume of water (4.5 ml). Spontaneous liposome formation occurred as soon as the ethanolic solution came into contact with the aqueous phase. The liposome suspension was then kept under stirring by vortexing for 5 minutes at room temperature. The obtained liposomal suspension was stored at 20 °C.

### Liposome preparation (microfluidic methods)

#### “Cross-flow” chip (#chip1-MHF)

Liposomes were prepared by injecting a lipid mixture (PC 90G 90 mM DDAB 10 mM) dissolved in ethanol into the central channel of the microfluidic network of #chip1-MHF; water was injected into two oblique side channels intersecting with the central channel. The flow rate ratio (FRR) is defined here as the ratio between the water volumetric flow rate and the ethanol volumetric flow rate. The FRR was varied from 10 to 50. Liposome formation at different shear forces was investigated by changing the total flow rate (TFR) from 18.75 to 75.00 μl/min. The flow focusing was observed and monitored with a Dino-Eye Eyepiece camera (Dino-lite Digital Microscope).

#### “Y-junction” chip (#chip2-YJ)

Liposomes were prepared *via* a similar procedure to that applied for the #chip1-MHF apart from the fact that the lipid mixture (PC 90 mM and DDAB 10 mM) dissolved in ethanol was injected into one channel of the Y geometry, while water was injected into the other channel. TFR was set between 18.75 and 75.00 μl/min, and FRR was varied from 10 to 50.

#### “Three-inlets pillars” chip (#chip3-PM)

The preparation of liposomes by the “Three-inlets pillars” chip (#chip3-PM) was analogous to the procedure described for “cross-flow” (#chip1-MHF). The TFR was varied from 18.75 to 75.00 μl/min and the FRR was varied from 10 to 50.

#### Off the shelf chip (#chip4-OFF3)

Microchip constituents were obtained from IDEX Health & Science, USA. #chip4-OFF3 components were made from PEEK and specifically designed with a 0.006” thru-hole that delivers a low swept volume and includes F-112 and P-416 fittings. To connect the “off-the-shelf” devices to the syringes, Teflon^TM^ tubes with an inner diameter of 750 μm were employed. KDS syringe pumps (KD scientific, New Era pump System, USA) were used to control the flow rate of liquids pumped through the devices.

#### Large channel MHF chip (#chip5-MHF-LC)

Liposomes were again prepared using a similar procedure to that above for #chip1-MHF apart from the fact that the central ethanol stream contained DMPC, Cholesterol and 14:0 PEG2000 PE at a concentration of 10, 8 and 2 mM/L (30 mM, 20 mM and 6 mM), respectively. The TFR was varied from 3 to 18 ml/min and the FRR was varied from 5 to 100.

### Physical and chemical characterization

#### Dynamic light scattering (DLS) analysis

All dimensional analysis of the liposome dispersions were performed using a DLS Zetasizer Nano-ZS (Malvern Instruments, Worcs., UK) with a backscattering detection angle of 173°, a He/Ne laser that emits at 633 nm, and a 4.0 mW power source was used to report the intensity mean diameter (Z-average) and the dispersity of the liposome formulations. The mean particle size was obtained from the results of three experiments. The size distribution was evaluated in terms of the dispersity index (Đ). The measurements of vesicle size and dispersity were carried out at 21 °C in water, without dilution (1 ml).

#### Cryogenic transmission electron microscopy (cryo-TEM)

A 3 mL aliquot of sample solution was pipetted on to plasma-treated (Gatan Solarus Model 950 Advanced Plasma System, p = 70 mTorr, H_2_ flow 6.4 sccm, O_2_ flow 27.5 sccm, orward RF target 50 W, time 30 s) carbon copper grids (Quantifoil R 3.5/1) in the environmental chamber of a fully automated vitrification device for plunge freezing (FEI Vibrot), having relative air humidity of 100% and a temperature of 22 °C. The excess solution was removed by blotting with filter paper for 2 s followed by 1 s draining and plunging of the samples into 1:1 mixture of liquid ethane and liquid propane, which was cooled to −170 °C. Vitrified samples were cryo-transferred into a Jeol JEM3200FSC cryo-TEM operating at −194 °C. The temperature of the samples was −187 °C during image acquisition. The microscope was operated in the bright field mode, using a 300 kV acceleration voltage; the in-column energy filter was set to 0–20 eV energy-loss range (zero-loss imaging). Micrographs were recorded with a Gatan Ultrascan 4000 CCD camera.

#### Determination of drug entrapment efficiency

The determination of drug entrapment efficiency in liposomes was performed by loading 250 μl of a liposome dispersion into a Sheparose^®^ 4B column (1.0 cm in diameter and 10 cm long; GE Healthcare, USA), eluted with Isotonic Palitzsch buffer. The void volume peak fractions containing drug loaded liposomes were collected and quantitated for drug content. UV-VIS measurement (at 253 nm) was conducted by diluting 300 μl of each fraction with 700 μl of ethanol. UV spectra were recorded with a Hewlett-Packard 8452 diode array spectrophotometer. Drug encapsulation efficiency (EE) was calculated as follows:


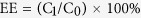


where C_0_ and C_1_ correspond to the total and liposome-associated amount of drug, respectively.

## Additional Information

**How to cite this article**: Carugo, D. *et al.* Liposome production by microfluidics: potential and limiting factors. *Sci. Rep.* **6**, 25876; doi: 10.1038/srep25876 (2016).

## Supplementary Material

Supplementary Information

## Figures and Tables

**Table 1:** Key features of liposomes present in commercial medicines.

**Composition**	**Concentration**^a^ **(mM)**	**Size**^b^ **(nm)**	**Manufacturing method**	**Physical form**	**Other excipients**
Phospholipids (mainly PC) and cholesterol	8 (mean) 2–17 (range) 0.1–0.2 (for vaccines)	80–100	Extrusion followed by active loading	Liquid suspension or lyophilized powder	pH adjusting agents Antioxidants Osmotic agents

^a^Total concentration of lipids in the liposomal formulation ready to be administered.

^b^Generally not indicated in the prescribing information leaflets or in the summary of product characteristics.

**Table 2:** Microfluidic procedures for the preparation of liposomes.

**Microchip material**	**Microchip geometry**^**a**^ **and dimensions (angle/depth/width/length) (°/**μ**m)**	**Lipids concentration (mM)**	**Lipid solvent**	**Water phase**	**TFR (**μ**l/min) FRR**	**Investigated experimental parameters**	**Procedure for liposome analysis (Z average)**	**ref.**
Silicon wafer	MHFn.i./100/42 or 64/n.i.	DMPC 2.5Ch 2.0DHP 0.5	IPA	PBS	*Experiment #1*TFR 31–186FRR 30*Experiment #2*TFR 22–122FRR10, 15, 20, 30, 40, 60	TFRFRR	AF4, MALLS, QELS	7
Silicon wafer	MHF*chip#1*45°/120/42 or 65/10000*chip#2*90°/36/10/10000	DMPC 2.5Ch 2.0DHP 0.5	IPA	PBS	*Experiment#1 (chip#1)*TFR 117FRR 12, 17, 24, 36, 48*Experiment #2 (chip#2)*TFR 5.4FRR 6,9,12,24,36*Experiment #3 (chip#1)*TFR 25, 50, 100FRR 14, 19, 29, 49	TFR FRR	AF4, MALLS, QELS	8
Silicon wafer	MHF90°/39 ± 1/21 ± 1/n.i.	DPPC 4.1Ch 1.19DCP 0.69	IPA	PBSNIPAMBADEAP	TFR 9.6FRR 10, 15, 20, 25	FRR	AF4, MALLS, 90Plus/BI-MAS	9
PDMS	MHF90°/50/200/5000	*Lipid phase #1*DMPE 2.5Ch 2.0DHP 0.5*Lipid phase #2*E. Coli PE 5.0	IPA	PBS	TFR 50–133FRR 2, 3, 4, 5, 6, 7	FRR C_comp_	Nano ZS90, angle 90°	10
Glass wafer	MHF90°/50/220/n.i.	*Lipid phase #1*POPC 2.0 –20.0*Lipid phase #2*DMPC 2.0–20.0	IPA	PBS	*Experiment#1*TFR 30–246FRR 4, 10, 20, 30, 40*Experiment#2*TFR66FRR10	FRR C_lip_ C_comp_	Nano ZS, angle 173°	11
PDMS	*Chip #1*n.i./100/140/n.i.single hydrodynamic focusing (SHF)*Chip #2*n.i./100/140/n.i.double hydrodynamic focusing (DHF)	EPC 12.5 – 37.5DOPE 6.25 – 18.75DOTAP 6.25 – 18.75	EA	water	TFR 80–160 mm/sFRR 8, 10, 13, 16, 18	FRR TFR C_lip_	Nano ZS, angle 173°	12
COC	MHFn.i./270/190/n.i.	*Lipid phase #1*DMPCChDCPPEG_5000_-PE(C_lip_ 20.0)*Lipid phase #2*DMPCChDCPPEG_2000_-PE(C_lip_ 20.0)	EA	BS	TFR 384FRR 40, 70, 100	FRR C_lip_	AF4, MALLS, QELS	13
PDMS-glass	MHFn.i./150/15/n.i.	DPPC 7.5Ch 6.0PEG2000-PE 1.5	EA	PBS	TFR 0.20 m/s (27)FRR 5, 7, 9, 10, 12	FRR	AF4, MALLS, QELS	14
PDMS	“Y” shape mixer with “herringbone“ elementsn.i./78/200/n.i.	*Lipid phase #1*DOPEDOTAP(8:8 μmol)*Lipid phase #2*PCCh(16:4 molar ratio PC/Ch)	EA	BS	*Experiment #1*TFR 500 – 2000FRR 1, 3, 5*Experiment #2*TFR 2000FRR 1, 3, 5*Experiment #3* TFR2000–6000 FRR3	TFRFRR	Nano ZS,angle 173°	15,16

^a^Geometry: angle = intersection angle between the central and side channels; depth = the average depth of the microfluidic channels; width = the average width of the microfluidic channels; length = the length of the main channel. Abbreviation: n.i. = not indicated; DMPC = 1,2-dimyristoyl-sn-glycerol-3-phosphatidylcholine; Ch = Cholesterol; DHP = dihexadecyl phosphate; IPA = isopropyl alcohol; PBS = phosphate buffered saline; TFR = total flow rate; FRR = flow rate ratio; AF4 = asymmetric flow field-flow fractionation; MALLS = multiangle laser light scattering; QELS = quasi-elastic light scattering; DPPC = 1,2-dipalmitoyl-sn-glycero3-phosphatidylcholine; NIPA = N-isopropylacrylamide¸MIPA = N,N 0 –methylenebis (acrylamide); DEAP = 2,2-diethoxyacetophenone; PDMS = polydimethylsiloxane; DMPE = 1,2-dimyristoyl-sn-glycerol-3-phosphatidylethanolamine; E. Coli PE = Escherichia coli phosphatidylethanolamine (containing phosphatidylethanolamine as major constituent and phosphatidyl glycerol, phosphatidic acid and cardiolipin); C_comp_ = total lipid concentration; POPC = 1-palmitoyl-2-oleyl-sn-glycerol-3-phosphatidylcholine; C_lip_ = total lipid concentration; EPC = egg phosphatidylcholine; DOPE = 1,2-dioleoyl-sn-glycero-3-phosphoethanolamine; DOTAP = 1,2-dioleoyl-3-trimethylammonium-propane; EA = ethyl alcohol; COC = cyclic olefin copolymer; DCP = dihexadecyl phosphate; PEG_5000_ = PE 1,2-dimyristoyl-sn-glycero3-phosphoethanolamine¸PEG_2000_ = PE 1,2-dimyristoyl-sn-glycero3-phosphoethanolamine-N-[methoxy(PEG)-2000]; BS = buffer solution; PC = phosphatidylcholine; MHF = microfluidic hydrodynamic focusing; ZS (or ZS90) = ZetaSizer.
