# Supplementary material for: Liposome production by microfluidics: potential and limiting factors
Source: Sci Rep. 2016 May 19;6:25876. doi: 10.1038/srep25876 (PMC4872163; doi:10.1038/srep25876)
Supplement: Supplementary Information [file srep25876-s1.pdf]

Supplementary Information for:

Liposome production by microfluidics: potential and limiting factors

Dario Carugo[1,2], Elisabetta Bottaro[3], Joshua Owen[1], Eleanor Stride[1] and Claudio Nastruzzi*[3]

[1]Institute of Biomedical Engineering, Department of Engineering Science, Old Road Campus Research Building, University of Oxford, Oxford, United Kingdom

[2]Faculty of Engineering and the Environment, University of Southampton, Southampton, UK

[3]Department of Life Science and Biotechnology, University of Ferrara, Italy

*Correspondence to:
Prof Claudio Nastruzzi, PhD
Department of Life Sciences and Biotechnology
University of Ferrara
via Fossato di Mortara 17, 44121, Ferrara, Italy
Phone: +39-0532-455348
e-mail: nas@unife.it
Web: www.biomaterials.it

Table S1 | Medicinal products containing liposomes (currently on the market)

**product name/ drug/ formulation**	**composition**	**lipid composition (mg/ml)**		**diluent**	**route of injection**
**Abelcet**<br>Amphotericin B<br>Suspension	*Lipids*<br>*DMPC, DMPG*<br>*Other excipients*<br>Sodium Chloride	*Starting*<br>*DMPC 5.01*<br>*DMPG 2.14*<br>Tot=7.15	*Ending*<br>*DMPC 3.4*<br>*DMPG 1.5*<br>Tot=4.9	After mix withdraw the correctly dose and dilute to 1 mg/ml with dextrose 5%	Intravenous
**Ambisome**<br>**Amphocil**<br>Amphotericin B<br>Powder	*Lipids*<br>HSPC,DSPG, chol, α-Toc, DSH<br>*Other excipients*<br>Sucrose, disodium succinate hexahydrate	*Starting*<br>HSPC 23.35<br>Chol 11.19<br>DSPG 8.98<br>α-Toc 0.124<br>DSH 13.88<br>Tot=57.52	*Ending*<br>HSPC 5.83<br>Chol 2.79<br>DSPG 2.25<br>α-Toc 0.031<br>DSH 3.47<br>Tot=14.37	Dilute it in dextrose 5 %	Intravenous
**Curosurf**<br>Phospholipid fraction from porcine lung suspension	*Lipids*<br>*Phospholipid fraction from porcine lung*	Phospholipid fraction from porcine lung 80<br>Tot=80		No dilution needed	Intratracheal or intrabronchial
**DaunoXome**<br>Daunorubicin<br>Emulsion	*Lipids*<br>DSPC, chol,<br>*Other excipients*<br>Sucrose, glycine, calcium chloride	*Starting*<br>DSPC 28.16<br>chol 6.72<br>Tot=34.80	*Ending*<br>DSPC 14.08<br>Chol 3.36<br>Tot=17.44	Diluted 1:1 with 5% dextrose	Intravenous

**Depocyt**<br>Cytarabine<br>Suspension	*Lipids*<br>DOPC, triolein, DPPG<br>*Other excipients*<br>Sodium chloride	DOPC 5.7<br>Chol 4.4<br>Triolein 1.2<br>DPPG 1<br>Tot=12.30		No dilution needed	Intravenous
**DepoDur**<br>Morphine sulfate<br>Suspension	*Lipids*<br>HSPC,DSPG,DOPG, chol,<br>tricaprylin, triolein	Chol 3.3<br>Triolein 0.1<br>DOPC 4.2<br>DPPG 0.9<br>Tricaprylin 0.3<br>Tot=8.80		No dilution needed	Epidural
**Doxil**<br>**Lipo-dox Caelyx**<br>Doxorubicin<br>Suspension	*Lipids*<br>HSPC,DSPG, chol¸ MPEG–DSPE<br>*Other excipients*<br>Ammonium sulfate, sucrose,<br>histidine	*Starting*<br>HSPC 9.58<br>Chol 3.19<br>MPEG–DSPE 3.19<br>Tot=15.96	*Ending*<br>HSPC 1.72<br>Chol 0.57<br>MPEG–<br>DSPE 0.57<br>Tot=2.86	Diluted in 250 ml of 5%<br>dextrose	Intravenous
**Epaxal**<br>Inactivated<br>hepatitis Avirus<br>(strain RG-SB)<br>Suspension	*Lipids*<br>*Lecithin, cephalin*<br>*Other excipients*<br>Sodium chloride	Lecithin 0.08<br>Cephalin 0.02<br>Tot=0.10		No dilution needed	Intramuscolar
**Inflexal V**<br>Inactivated<br>hemaglutinine of<br>Influenza virus<br>strains A and B<br>Suspension	*Lipids*<br>Lecithin<br>*Other excipients*<br>Sodium chloride, disodium<br>phosphate dehydrate, otassium<br>dihydrogen phosphate	Lecithin 0.234<br>Tot=0.234		No dilution needed	Intramuscolar
**Marqibo**<br>Vincristine sulfate<br>suspension	*Lipids*<br>Sphingomyelin, chol<br>*Other excipients*<br>Mannitol, sodium citrate, sodium<br>phosphate, sodium chloride	Sphingomyelin 73.5<br>Chol 29.5<br>Tot=103.0		The final drug product is<br>prepared on site from<br>the components in the<br>Marqibo Kit	Intravenous

**Myocet**<br>Doxorubicin<br>Powder	*Lipids*<br>EPC, chol<br>*Other excipients*<br>Citric acid, sodium hydrixide	EPC<br>Chol<br>(55:45 molar ratio)	Withdraw 20 ml sodium chloride solution for injection (0.9%), and inject into Myocet doxorubicin HCl, intended for preparation	Intravenous
**Visudyne**<br>Verteporfin<br>Powder	*Lipids*<br>HSPC,DSPG, chol<br>ascorbyl palmitate<br>*Other excipients*<br>Butylated hydroxytoluene	EPC<br>DMPC<br>(3:5 molar ratio)	Reconstitute Visudyne with 7.0 ml water for injections to produce 7.5 ml of a 2.0 mg/ml solution	Intravenous

Abbreviation: **HSPC = hydrogenated soy phospatidylcholine; DSPG = distearoylphospatidyl glycerol; chol = cholesterol;α-Toc = alpha–tocopherol; DSH = disodium succinate hexahydrate; DMPC = dimyristoylphosphatidylcholine; DMPG = dimyristoylphosphatidylglycerol; DSPC = distearoylphospatidylcholine ; MPEG–DSPE = N-(carbonyl-methoxypolyethylene glycol 2000)-1,2-distearoyl-sn-glycero-3-phospho-ethanolamine sodium salt ;EPC = egg phosphatidylcholine; DOPC= dioleoylphosphatidylcholine; DPPG = dipalmytoilphospatidylglycerol; DOPG = dioleoylphosphatidylcholine**

Date were obtained from the information leaflets included into the packaging of the reported medicines.
Leaflets can be consulted at the following websites.

Abelcet: www.medicines.org.uk/emc/PIL.28850.latest.pdf
Ambisome: www.medicines.org.uk/emc/PIL.8363.latest.pdf
Amphocil: www.infarmed.pt/infomed/download_ficheiro.php?med_id=360&tipo_doc=fi
Curosurf: www.medicines.org.uk/emc/PIL.21480.latest.pdf
DaunoXome: www.medicines.org.uk/emc/PIL.25594.latest.pdf
Depocyt: www.medicines.org.uk/emc/PIL.15024.latest.pdf
DepoDur: www.mhra.gov.uk/home/groups/spcpil/documents/spcpil/con1428643391518.pdf
Doxil: www.doxil.com/shared/product/doxil/doxil-prescribing-information.pdf
Lipo-dox Caelyx: www.janssen.com/canada/sites/www_janssen_com_canada/files/product/pdf/cae10102013cpm_nc.pdf
Epaxal: www.medicines.org.uk/emc/PIL.26278.latest.pdf
Inflexal V: www.medicines.org.uk/emc/PIL.24908.latest.pdf?isAttachment=true&documentid=24908
Marqibo: www.accessdata.fda.gov/drugsatfda_docs/label/2012/202497s000lbl.pdf
Myocet: www.tevauk.com/mediafile/id/20670.pdf
Visudyne: www.medicines.org.uk/emc/PIL.4647.latest.pdf

Table S2 │ Volumetric ratio and relative volumes for water, ethanol and lipid solution in ethanol employed in the bulk, controlled ethanol injection experiments.

Variation	Vr = 10			Vr = 20			Vr = 30			Vr = 40			Vr = 50		
	**L**	**Et**	**W**	**L**	**Et**	**W**	**L**	**Et**	**W**	**L**	**Et**	**W**	**L**	**Et**	**W**
Lipids	455	-	4545	238	217	4545	162	293	4545	122	333	4545	98	357	4545
Et	98	357	4545	98	140	4762	98	64	4838	98	24	4878	98	-	4545
Lipids Et	455	-	4545	238	-	4762	162	-	4838	122	-	4878	98	-	4902

Abbreviation: Vr = volumetric ratio between water and the ethanolic lipid solution; L = lipid solution; Et= ethanol; W = water. Volumes are expressed in microliters.
